# Supplementary material for: Misalignment with the external light environment drives metabolic and cardiac dysfunction
Source: Nat Commun. 2017 Sep 12;8:417. doi: 10.1038/s41467-017-00462-2 (PMC5595905; doi:10.1038/s41467-017-00462-2)
Supplement: Supplementary file 1 — Supplementary Information [file 41467_2017_462_MOESM1_ESM.pdf]

Description of Supplementary Files

File name: Supplementary Information
Description: Supplementary figures and supplementary table.

File name: Peer review file

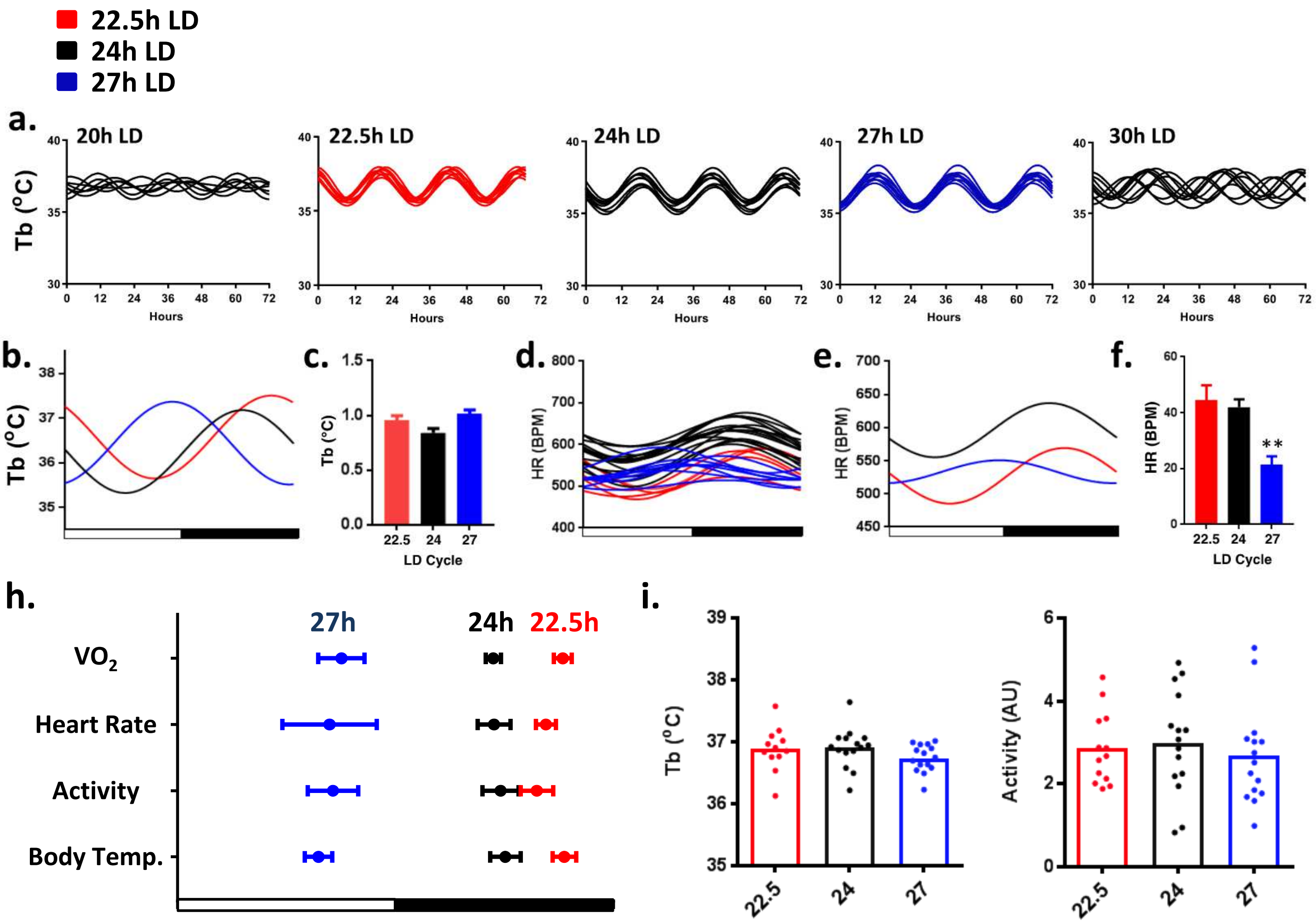

Supplementary Figure 1: Phase synchrony is maintained between animals and between physiological rhythms despite alterations in period and phase of entrainment in 22.5h and 27h LD housed mice. a. Individual mouse T_b waveforms from animals housed under 20-30h LD cycles (sine-wave fit by regression analyses). **b.** Group average $T_b$waveform profile (10d recording/animal). **c.** T_b rhythm amplitude derived from the regression analyses. **d.** Individual mouse heart rate (HR) waveform profiles. **e.** Group average HR rhythm waveform. **f.** HR rhythm amplitude was significant reduced in the 27h LD housed group (derived from sinusoidal waveform fit). ** P<0.01 versus 24h LD one-way ANOVA with Dunnet's post hoc test. **h.** Acrophase of physiological and behavioural rhythms under 24h and non-24 hr LD cycles. Although the mice exhibit a significant advance (27h) or delay (22.5) in acrophase, consistent phase was maintained between physiological parameters. **i**. No significant difference was observed in Tb or activity levels in 24h and non-24h LD housed mice (recorded over 7d).

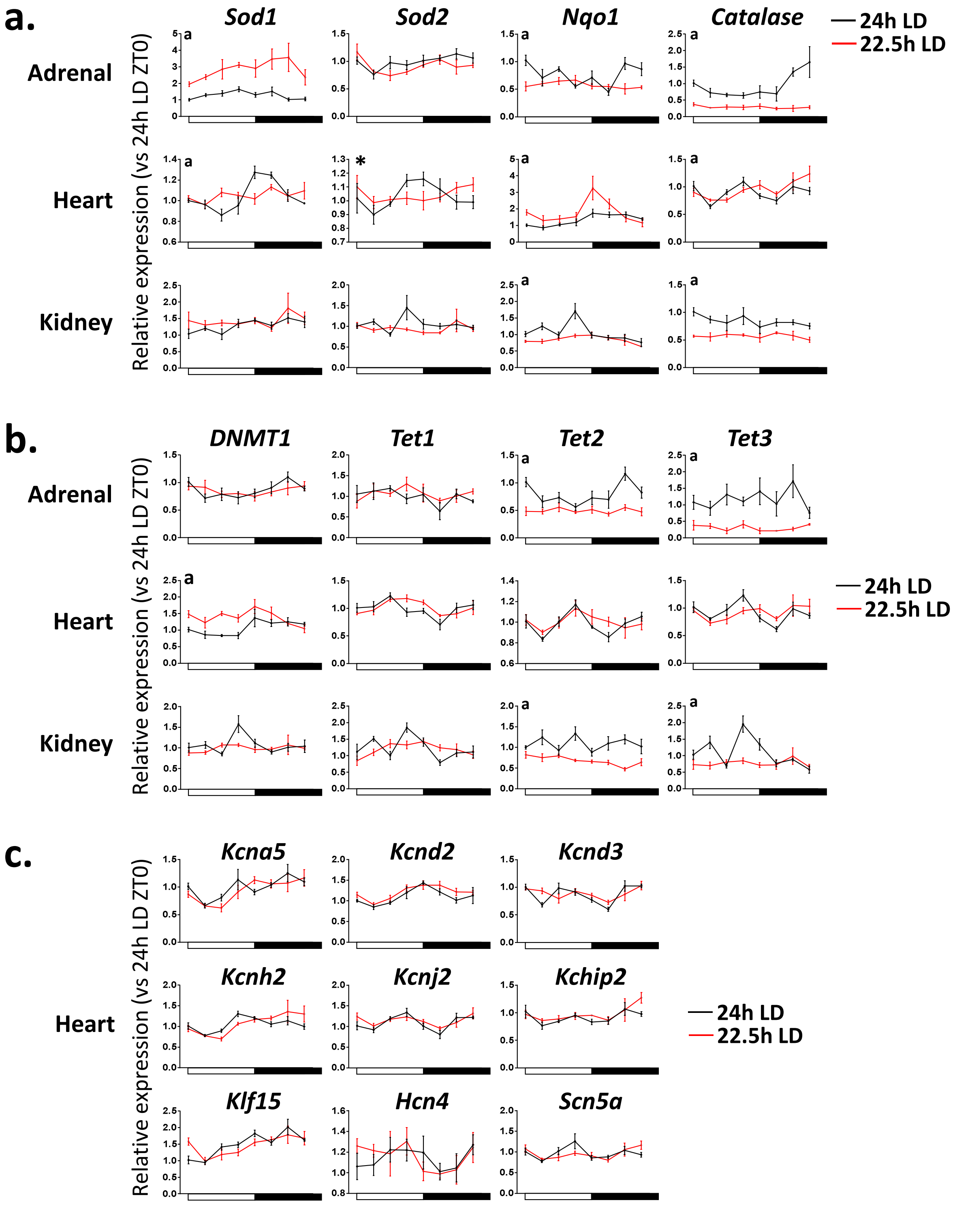

Supplementary Figure 2: Impact of 22.5h LD cycles on gene expression. Diurnal profiles in mRNA expression of (**a**) antioxidant pathways and (**b**) methylation machinery in adrenal gland, heart and kidney. (**c**) Diurnal profile of ion channel expression in cardiac tissue. All data plotted mean +/- SEM relative to the respective light cycle (24 or 22.5h) and normalized to ZT0 of the 24h LD group (n=4/time-point/group). * = significant ($P<0.05$) difference in phase between 24h and 22.5h profiles; a = significant difference in amplitude or mesor between 24h and 22.5h LD conditions (sinusoidal waveform fits with F tests for shared characteristics).

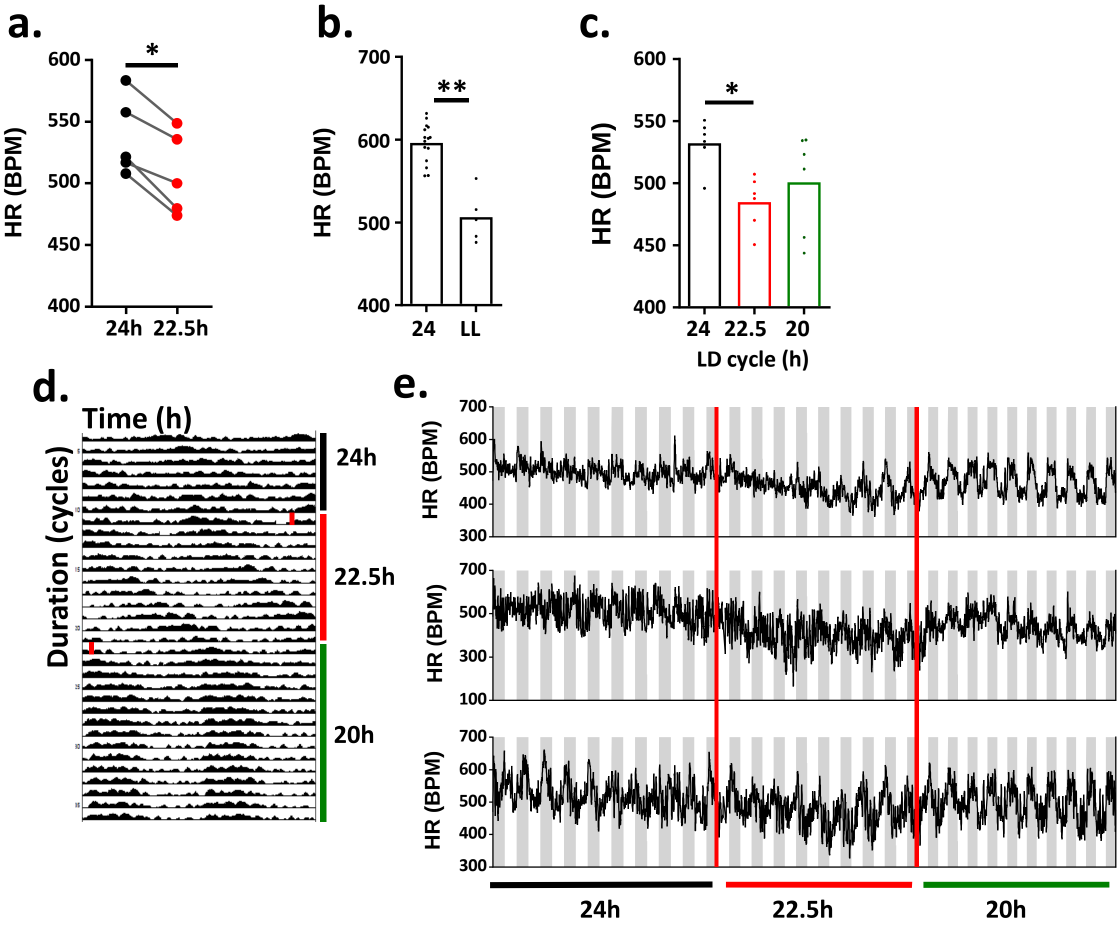

Supplementary Figure 3: Rapid depression of heart rate by aberrant lighting environment. a. Decrease in HR was observed rapidly upon transferring the mice from 24h LD (black) to 22.5h LD cycles (red)(*P<0.05 paired T-test; HR measured over 5days pre- and post- LD switch respectively). **b.** Mean HR of mice maintained in 24h LD cycles or two weeks of constant light (LL) exposure (*P<0.05, T-test). **c.** HR recordings from *CK1ε*^{tau} mutant mice across 24h, 22.5h, and 20h LD cycles. **d.** Representative Tb profile from CK1ε^{tau} mutant mice across shortening LD cycle length**. e.** representative longitudinal HR recordings from *CK1ε*^{tau} mutant mice maintained on shortening LD cycles. Grey bars indicate dark periods, white bars represent light periods. *p<0.05, repeated-measures one-way ANOVA with Tukey's post-hoc test.

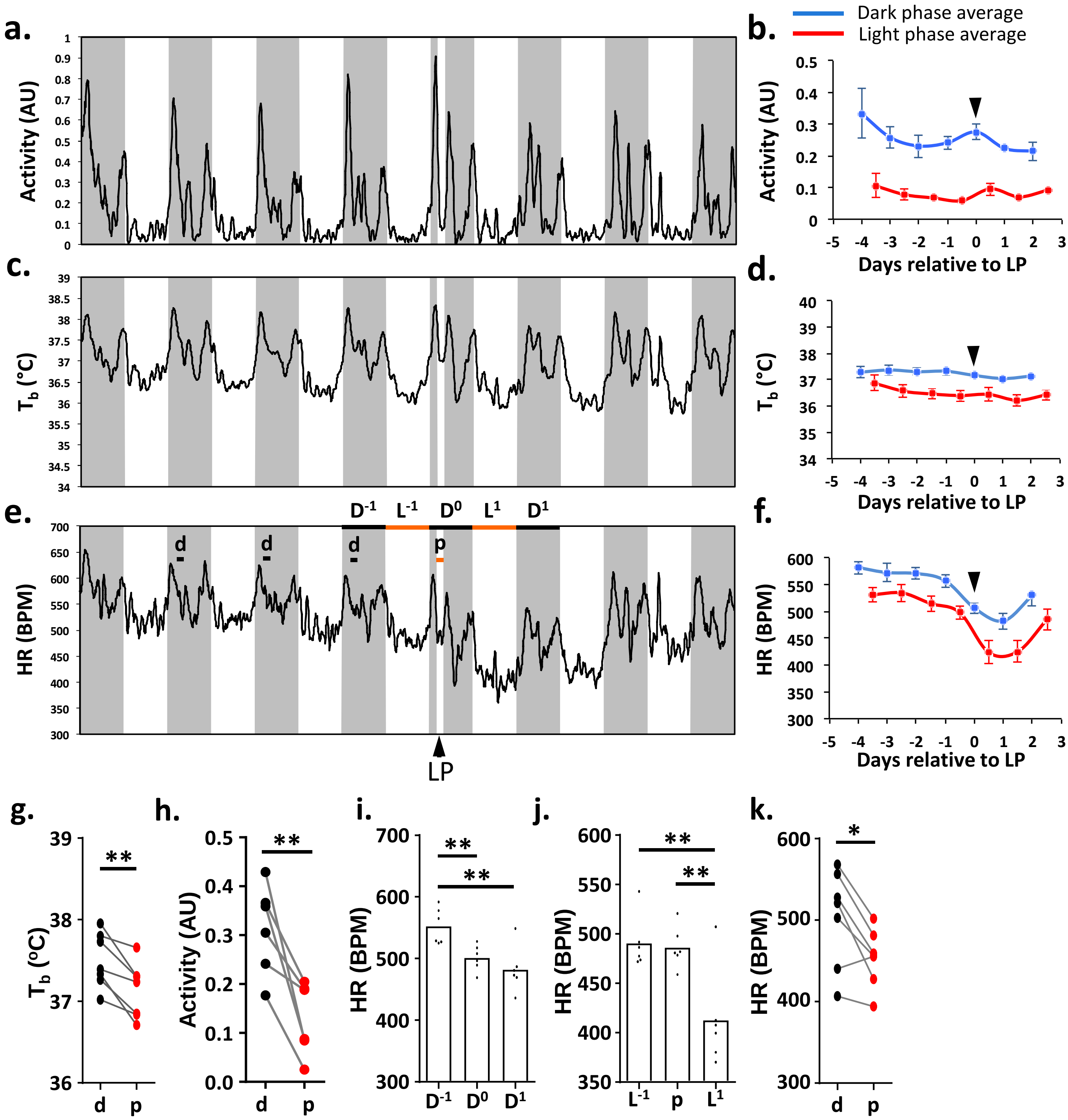

Supplementary Figure 4: Impact of acute light pulse on heart rate (HR) dynamics. a-f. Recording of activity (**a**) body temperature (**c**) and HR (**e**) prior to and after a 2h light pulse from ZT14-16 (LP, marked with arrowhead; traces depict group mean; n=7/group). **b, d, e.** Mean activity (**b**), T_b (**c**) and HR (**e**) across the light and dark phase of each cycle preceding, during and following the light pulse. **g.** Mean T_b across ZT14-16 during the pulse (red, p) or the equivalent period across the preceding 3 cycles (black, d). **h.** Mean locomotor activity across ZT14-16 during the pulse (red, p) or the equivalent period across the preceding 3 cycles (black, d). **i.** Mean HR across the entire dark phase prior to, during and following the LP. **H.** Mean HR across the light phase prior to, during and following LP. **i**. HR across ZT14-16 prior to and during LP when limited to inactive periods (no activity recorded for 20 minutes prior to HR measure). D^{-1} = night prior to the LP, D^0 = night of the LP, D^1= night after the LP, L^{-1} = light phase prior to the LP, L^1 = light phase after LP, d = ZT14-16 average across the 3 LD cycles preceding the LP. **g**, **h**, **k**, *p<0.05, **p<0.01 paired T-test; **i**, **j**. **p<0.01 repeated-measures one-way ANOVA with Tukey's post-hoc test.

Gene Name	Forward 5'-3'	Reverse 5'-3'
Ppib	GGA GAT GGC ACA GGA GGA AA	CCG TAG TGC TTC AGT TTG AAG TTC T
r18s	TCC GAC CAT AAA CGA TGC CGA CT	TCC TGG TGG TGC CCT TCC GTC AAT
Bmal1	GGA CTT CGC CTC TAC CTG TTC A	AAC CAT GTG CGA GTG CAG GCG C
Per2	GCC TTC AGA CTC ATG ATG ACA GA	TTT GTG TGC CTC AGC TTT GG
Cry1	TCG CCG GCT CTT CCA A	TCA AGA CAC TGA AGC AAA AAT CG
Nr1d1	GTC TCT CCG TTG GCA TGT CT	CCA AGT TCA TGG CGC TCT
Dbp	CCG TGG AGG TGC TAA TGA CCT	CCT CTG AGA AGC GGG CC
Pparg	AGG CCG AGA AGG AGA AGC TGT TG	TGG CCA CCT CTT TGC TCT GCT C
Wee1	GAA ACA AGA CCT GCC AAA AGA A	GCA TCC ATC TAA CCT CTT CAC AC
Nrf2	CTC GCT GGA AAA AGA AGT G	CCG TCC AGG AGT TCA GAG G
RelA	TGT GGA GAT CAT CGA ACA GCC G	TTC CTG GTC CTG TGT AGC CAT TGA T
Ezh2	GGG CTA TCC AGA CTG GTG AA	AAG GCA GCT GTT TCA GAG AGA
Kcn5a	CCT GCC CCG CAA TGA G	ACG ATG GCA ATG GCT CTT G
Kcnd2	TCG CCC ATC AAG TCA CAG TC	CTG GAG GTG TTG GGA TGC TT
Kcnd3	TCA CAA GCA TCC CTG CAT CTT	CAA ATA TCT TCC CTG CGA TTG TC
Kcnh2	GAT CGC CTT CTA CCG GAA A	CAT TCT TCA CGG GTA CCA CA
Kcnj2	TCC CTC CCT TTC CCA AAC AC	GAG GCT TGA TTT TGA GAC GC
Kchip2	AGC GTG GAG GAT GAG TTT GAA C	TTC CCC GAA GAA TCA CTG ACA
Klf15	CAA GAG CAG CCA CCT CAA G	GAC ACT GGT ACG GCT TCA CA
Hcn4	GCA TGA TGC TTC TGC TGT GTC ACT	TTC ACC ATG CCA TTG ATG GAC ACC
Scn5a	AGA CTT CCC TCC ATC TCC AGA TA	TGT CAC CTC CAG AGC TAG GAA G
DNMT1	CCT AGT TCC GTG GCT ACG AGG AGA A	TCT CTC TCC TCT GCA GCC GAC TCA
Tet1	GAA GGA ACA GGA AGC TGC AC	CTG GCC AAA CCT AGT CTC CA
Tet2	GAT CCA GGA GGA GCA GTG AG	TGG GAG AAG GTG GTG CTA TC
Tet3	CCG GAT TGA GAA GGT CAT CTA C	AAG ATA ACA ATC ACG GCG TTC T

Supplementary Table 1. Forward and reverse primer sequence used for qPCR
